# Wnt signaling contributes to vascular calcification by induction of matrix metalloproteinases

**DOI:** 10.1186/s12872-016-0362-8

**Published:** 2016-09-30

**Authors:** Christian Freise, Nadja Kretzschmar, Uwe Querfeld

**Affiliations:** 1 Center for Cardiovascular Research, Charité - University Medicine, Campus Mitte, Hessische Str. 3-4, 10115 Berlin, Germany; 2 Department of Pediatric Nephrology, Charité - University Medicine, Campus Virchow Clinic, 13353 Berlin, Germany

**Keywords:** Matrix metalloproteinases, Vascular calcification, Chronic kidney disease, Vascular smooth muscle cells, Wnt pathway, MOVAS-1

## Abstract

**Background:**

Vascular calcifications such as arteriosclerosis, which is characterized by a calcificiation of the *tunica media*, represent major comorbidities e.g. in patients with chronic kidney disease (CKD). An essential step during the development of arteriosclerosis is the transdifferentiation/calcification of vascular smooth muscle cells (VSMC) resembling osteogenesis. The matrix metalloproteinases (MMP)-2 and −9 were shown to promote these VSMC calcifications and their inhibition is of therapeutic value to prevent arteriosclerosis in preclinical studies. Aiming for an understanding of the underlying regulatory mechanisms of MMPs we here investigated, if the MMP-mediated VSMC calcification involves altered signaling of the Wnt pathway, which is known to impact osteogenesis.

**Methods:**

We used an experimental in vitro model of vascular calcification. Transdifferentiation/calcification of murine VSMC was induced by elevated calcium and phosphorus levels. Calcification was assessed by calcium and alkaline phosphatase measurements. Activation/activity of the gelatinases MMP-2 and MMP-9 was assessed by conversion of fluorescence-labelled substrates. Activation of the Wnt pathway was analysed by a reporter gene assay.

**Results:**

Besides pro-calcifying culture conditions, also activation of Wnt signaling by a specific agonist (under normal culture conditions) stimulated VSMC-calcification accompanied by enhanced expression and secretion of the gelatinases MMP-2 and −9. Vice versa, recombinant MMP-2 and −9 induced a time-delayed activation of Wnt signaling after 72 h in VSMC but showed no direct effects after 24–48 h. These effects were blocked by pharmacological inhibition of MMPs or of Wnt signaling.

**Conclusions:**

Our study suggests that the pro-calcifying environment in CKD induces Wnt signaling in VSMC which in turn contributes to the induction of MMPs which then foster the development of arteriosclerosis. Thus, besides MMP inhibition, the inhibition of Wnt signaling in VSMC might represent a therapeutic target for the prevention of vascular calcifications.

**Electronic supplementary material:**

The online version of this article (doi:10.1186/s12872-016-0362-8) contains supplementary material, which is available to authorized users.

## Background

Vascular calcifications are a clinical hallmark of advanced cardiovascular disease (CVD) in the general population. Besides chronic kidney disease (CKD) also hypertension, diabetes mellitus, dyslipidaemia, obesity and smoking could contribute to the development of vascular calcifications [1]. Calcifications are associated with increased cardiovascular morbidity and mortality from CVD, creating a major public health burden [2–5]. A well-known type of calcification is atherosclerosis which is characterized by intimal plaque formation and which could lead to infarction or ischemia. A second type of calcification is arteriosclerosis or mönckeberg’s sclerosis. Arteriosclerosis is characterized by calcification of the *tunica media* which amongst others could result in vessel stiffness and “pseudo” hypertension [6]. Studies suggest that the mortality of CKD patients is linked more to arterial stiffening, a consequence of medial calcification, than to atherosclerosis [7]. The development of arteriosclerosis is based on a process of biomineralization resembling osteogenesis [8, 9]. This involves an extensive remodelling of the arterial extracellular matrix e.g. a changed composition of collagens, degradation of elastic fibres and transdifferentiation of vascular smooth muscle cells (VSMC) from a contractile to a chondroblast-like phenotype [10]. Proteolytic matrix metalloproteinases (MMPs) contribute to the remodelling of the extracellular matrix e.g. by degradation of elastic fibers and subsequent release of bioactive matrix fragments [11]. Further, MMPs were shown to exhibit also mitogenic activities on cells including VSMC [12–14] and to promote calcium and phosphorus (Ca/P)-induced transdifferentiation of VSMC in vitro [15]. Vice versa, inhibition of MMPs exerts protective effects against vascular calcification in experimental in vitro and in vivo models [15]. Thus, increased matrix degradation by MMPs could provide an early signal in the pathophysiology of calcifications and inhibition of MMPs could be of therapeutic value.

However, little is known about regulation of MMP secretion by VSMC especially in arteriosclerosis. Earlier studies on atherosclerosis demonstrated that the cleavage of cell surface N-cadherin by MMP-9 causes activation of the Wnt pathway in VSMC [16]. Interestingly, Wnt signaling is known to impact osteoblast development and to stimulate chondrogenic differentiation in pericytes [6]. Thus, regulation of cellular differentiation by Wnt/ß-catenin pathway is critical for vascular homeostasis [17].

Besides using specific Wnt agonists and antagonists, we therefore investigated whether stimulatory and inhibitory effects of recombinant MMPs and MMP inhibitors, respectively, involve altered Wnt signaling also in an in vitro model of arteriosclerosis based on Ca/P-induced VSMC calcification.

## Methods

### Cell culture

The murine VSMC cell line (MOVAS-1) was purchased from ATCC (ATCC® CRL-2797™) and cells were cultured in a humidified atmosphere at 37 °C and 5 % CO_2_. Standard culture medium consisted of DMEM with 862 mg/l L-alanyl-L-glutamine, 6 mmol/l glucose, 50 μg/ml streptomycin, 50 units/ml penicillin, 0.2 mg/ml G418, supplemented with 10 % heat-inactivated fetal bovine serum (FBS; Biochrom, Berlin, Germany).

### Induction of VSMC calcification

VSMC were grown in 12- or 24-well plates to ~90 % confluency (day 0). Calcification was induced by a calcification medium (CM) consisting of standard culture medium supplemented with NaH_2_PO_4_ and CaCl_2_ to final concentrations of PO_4_^3−^ (2.8 vs. 1.0 mmol/l) and Ca^2+^ (2.7 vs. 1.8 mmol/l), respectively. Cells were treated for up to 9 days as indicated and media were replaced every 2–3 days.

To study their effects on VSMC calcification, the recombinant MMPs-2 and −9 (3 nM; BioTez, Berlin, Germany) and the specific inhibitors for MMP-2 (MMP-2 inhibitor I; 10 μM; Merck Schwalbach, Germany), MMP-9 (MMP-9 inhibitor I; 1 μM; Merck) and both gelatinases (Ro28-2653; 1 μM; Roche Diagnostics GmbH, Penzberg, Germany) were added to CM.

### Quantification of VSMC calcification

The differently treated VSMC were decalcified with 0.1 N HCl for 30 min. Calcium contents were determined as described [18] by the o-cresolphthalein complexone method and normalized to respective protein contents.

Enzymatic activity of alkaline phosphatase (ALP) as a marker of VSMC calcification was assessed in VSMC supernatants using the Quanti Blue reagent (InvivoGen, San Diego, CA, USA) after 24 h of treatment. ALP activities were normalized to total protein contents.

### Quantitative real-time reverse transcription–polymerase chain reaction (RT–PCR)

To study effects of CM on Wnt5a expression and of the Wnt agonist I on MMP-2 and MMP-9 expression in VSMC, the cells were treated as indicated for 24 h and total RNA was isolated with the RNeasy Mini Kit (Qiagen, Hilden, Germany) and transcribed into cDNA with the High Capacity RNA to DNA kit (Applied Biosystems, Foster City, CA, USA). The cDNA concentrations were determined by a NanoDrop ND-1000 device (NanoDrop Technologies, Wilmington, NC). Quantitative PCR using SYBR Green Master Mix (Applied Biosystems) and subsequent melting curve analysis was performed using the Mx3000p system (Stratagene/Agilent Technologies, Waldbronn, Germany). Relative RNA amounts were calculated using the 2^−ΔΔCt^ method and normalized to mRNA expressions of the housekeeping genes YWAHZ (tyrosine 3-monooxygenase/tryptophan 5-monooxygenase activation protein, zeta polypeptide) and HPRT (hypoxanthine-phophoribosyl transferase). The primers sequences were: Wnt5a: forward primer, 5′-CAAATAGGCAGCCGAGAGAC-3′, reverse primer, 5′-CTCTAGCGTCCACGAACTCC-3′; MMP-2: forward primer, 5′-CAGGGAATGAGTACTGGGTCTATT-3′, reverse primer, 5′-ACTCCAGTTAAAGGCAGCATCTAC-3′; MMP9: forward primer, 5′-AATCTCTTCTAGAGACTGGGAAGGAG-3′, reverse primer, 5′-AGCTGATTGACTAAAGTAGCTGGA-3′ (BioTez Berlin-Buch GmbH, Berlin, Germany).

### Fluorogenic MMP activity assay

Treatment dependent enzymatic activities of MMP-2 and MMP-9 in VSMC supernatants were measured by cleavage of 0.01 mg/ml dye-quenched DQ-gelatin (Molecular Probes, Life Technologies GmbH, Darmstadt, Germany) as described in detail earlier [18].

To determine effects of the Wnt inhibitor on enzymatic activities of MMP-2 and MMP-9, recombinant MMP-2 and MMP-9 alone or mixed with the Wnt inhibitor FH535 (10 μM; CAS 108409-83-2; Merck, Schwalbach, Germany) were subjected to DQ-gelatin measurements.

### Transient transfection of VSMC and assessment of Wnt activation

VSMC were transiently transfected with reporter plasmids (Promega, Mannheim, Germany) for the T-cell factor (TCF)/lymphoid enhancer-binding factor (LEF) binding motif (pGL4.49[luc2P/TCF-LEF RE/Hygro]) and a renilla vector (pGL4.74[hRluc/TK]) as described [18]. As a positive control for Wnt activation the Wnt agonist I (CAS 853220-52-7; Merck, Schwalbach, Germany) was used. To inhibit Wnt activation, the β-Catenin/TCF site-inhibitor FH535 (10 μM; Merck) was used.

### Statistical analyses

Data were analyzed by one-way ANOVA followed by the Tukey post-hoc test using GraphPad PRISM, version 5.01 (GraphPad Software, San Diego, CA, USA) and differences with *P* values ≤0.05 (*) were considered to be statistically significant.

## Results

### CM-induced calcifications involve activation of Wnt signaling in VSMC

**Fig. 1:**
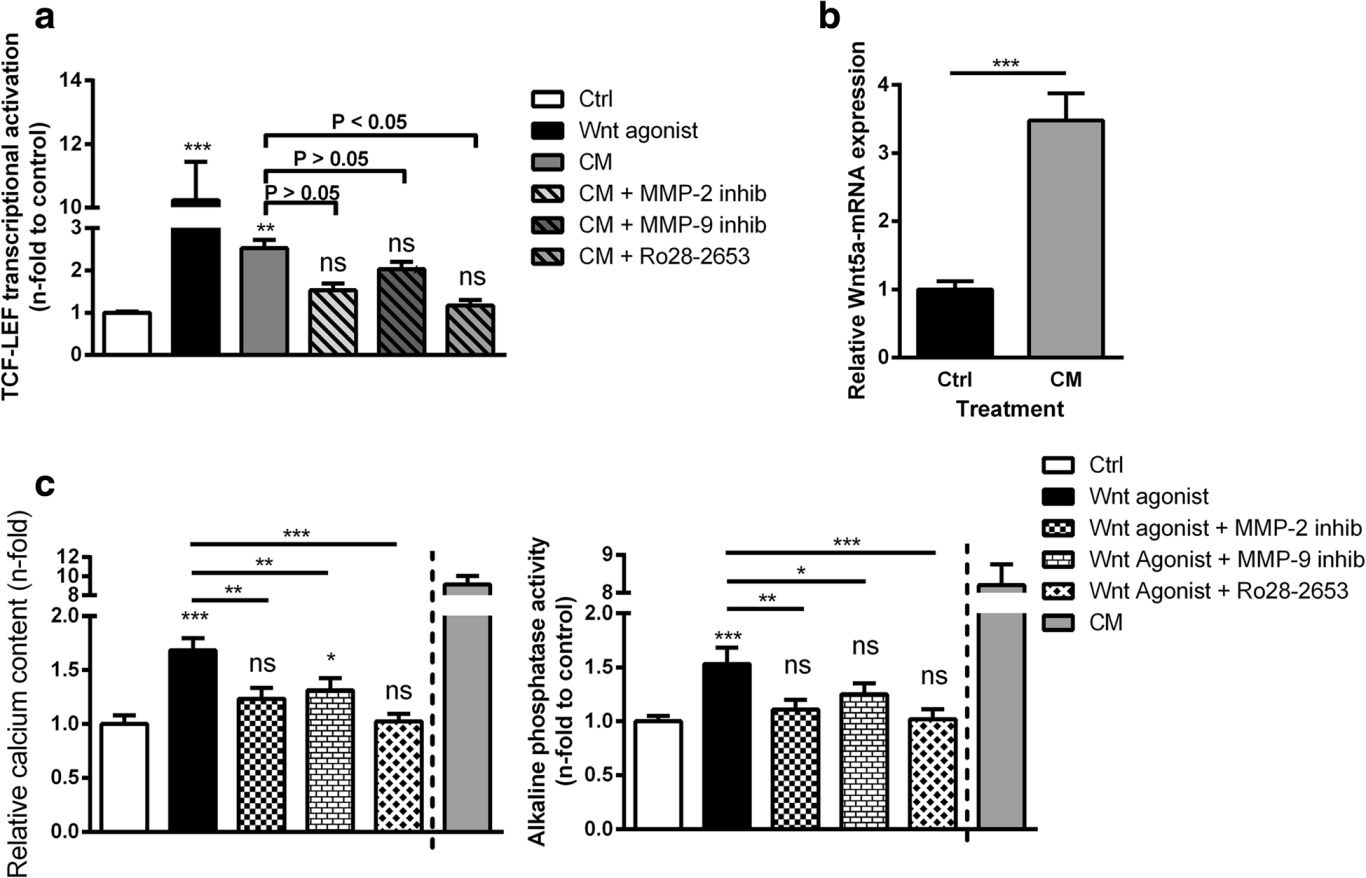
Effects of calcification medium, Wnt agonist I and gelatinase inhibitors on Wnt activation, Wnt5a mRNA expression and calcification of VSMC. **a** VSMC transfected with TCF/LEF-luciferase reporter plasmids were treated as indicated and luciferase activity as an indicator for activation of Wnt signaling was measured. Data were normalized to a parallel transfected renilla reporter vector (*n* = 4). **b** Effects of CM on the mRNA expression of Wnt5a were determined by qPCR measurements (*n* = 4) **c** VSMC were treated as indicated. Measurements of calcium contents or alkaline phosphatase (ALP) activities in VSMC cultures served as markers for VSMC calcifications (*n* = 4). Shown are means ± SD. ^ns^not significant to control, *P ≤ 0.05 (compared to control), **P ≤ 0.01 (compared to control), ***P ≤ 0.001 (compared to control)

In a first set of experiments we investigated whether a calcifying environment alters the activation of Wnt signaling in VSMC. As a positive control for Wnt activation, the transfected VSMC were treated with the Wnt agonist I which caused a 10-fold higher activation of Wnt signaling compared to control (Fig. 1a). Interestingly, also treatment with CM induced a 2.5-fold higher Wnt activation compared to control. Since the gelatinases were previously shown to be involved in VSMC calcification [15] and are also known to be upregulated by CM, we additionally applied specific inhibitors for MMP-2, MMP-9 or both gelatinases in our experiments. As shown in Fig. 1a, all inhibitors tend to reduce the CM-induced activation of Wnt signaling in VSMC but only simultaneous inhibition of both MMPs (Ro28-2653) provoked a significant reduction.

Direct effects of the MMP inhibitors on Wnt signaling were excluded by additional experiments showing no interference of the inhibitors with Wnt activation by the Wnt agonist (Additional file 1: Figure S1).

We additionally investigated the effects of CM on the mRNA-expression level of Wnt5a, a protein involved in Wnt signaling and calcification. As shown in Fig. 1b, CM-treated VSMC exhibited a 3.5-fold enhanced Wnt5a-mRNA expression compared to control.

### Wnt-induced calcifications of VSMC involve enhanced expression and secretion of MMP-2 and MMP-9

We next examined whether the stimulation of Wnt signaling in the absence of CM was sufficient to induce VSMC calcifications. Indeed, treatment of VSMC for 5 days with the Wnt agonist significantly induced elevated calcium contents and enzymatic ALP activities in VSMC cultures and VSMC supernatants, respectively. Both effects could be rescued most effectively by simultaneous inhibition of MMP-2 and MMP-9 (Fig. 1c).

**Fig. 2:**
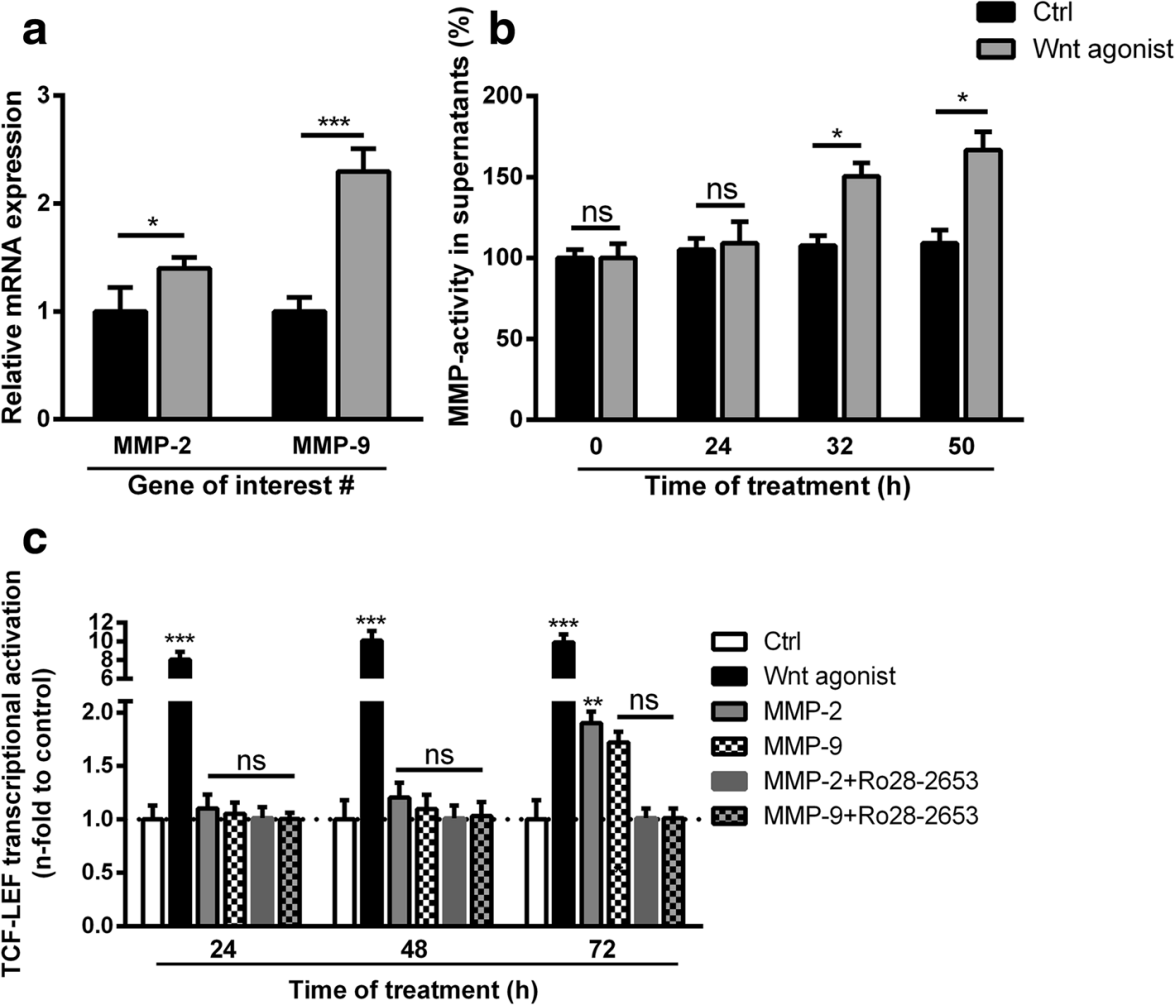
Effects of Wnt signaling on gelatinase gene expression and secretion and reverse effects of recombinant gelatinases on Wnt signaling. **a** VSMC were treated for 24 h with the Wnt agonist I and gene expressions of MMP-2 and MMP-9 were determined by qPCR measurements (*n* = 3). **b** VSMC were treated as indicated for up to 72 h. Gelatinolytic activities in aliquots of supernatants were determined by conversion of dye-quenched (DQ)-gelatin substrate at indicated time points (*n* = 4). **c** VSMC transfected with TCF/LEF-luciferase reporter plasmids were treated as indicated for 3 days and luciferase activity as an indicator for activation of Wnt signaling was measured. Data were normalized to a parallel transfected renilla reporter vector (*n* = 4). Shown are means ± SD; ^ns^not significant to control, *P ≤ 0.05 (compared to control), ***P ≤ 0.001 (compared to control)

Since the gelatinases seemed to be involved in the Wnt-mediated actions in VSMC, we consequently investigated effects of the Wnt agonist on the expression levels of MMP-2 and MMP-9 in VSMC. Indeed, treatment of VSMC with the Wnt agonist induced mRNA expressions of MMP-2 (1.4-fold) and MMP-9 (2.3-fold) (Fig. 2a) and also induced elevated gelatinolytic activities in VSMC supernatants (Fig. 2b).

Vice versa, we also checked whether recombinant MMP-2 and MMP-9 contribute to Wnt activation in VSMC. Treatment of VSMC with recombinant MMP-2 or MMP-9 had no effect on Wnt activation in VSMC during the first 24–48 h (Fig. 2c). However, a slightly induced Wnt activation in VSMC treated with recombinant MMP-2 was observed after 72 h which was blocked in the presence of the gelatinase inhibitor Ro28-2653 (Fig. 2c).

### Wnt agonist-induced effects can be blocked by specific Wnt inhibition via TCF-sites

**Fig. 3:**
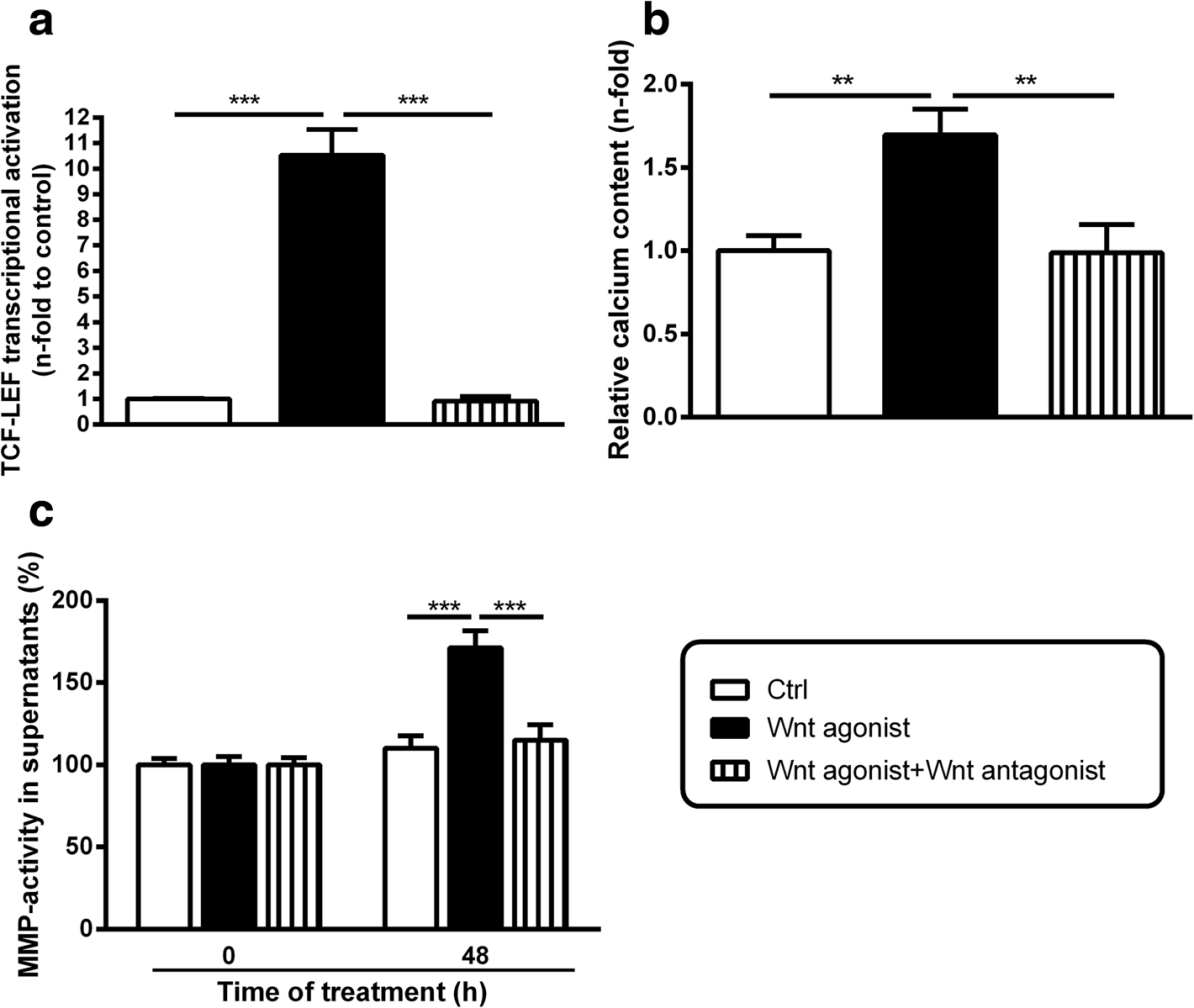
Effects of Wnt antagonism on Wnt agonist-induced Wnt activity, calcification and MMP-production in VSMC. **a** VSMC transfected with TCF/LEF-luciferase reporter plasmids were treated as indicated for 3 days and luciferase activity as an indicator for activation of Wnt signaling was measured. Data were normalized to a parallel transfected renilla reporter vector (*n* = 3). **b** VSMC were treated as indicated. Treatment dependent calcium contents of VSMC cultures were determined by the o-cresolphthalein complexone method. (*n* = 4). **c** VSMC were treated as indicated and gelatinolytic activities in aliquots of supernatants were determined by conversion of dye-quenched (DQ)-gelatin substrate (*n* = 3). Shown are means ± SD, **P ≤ 0.01, ***P ≤ 0.001

To investigate whether the Wnt-induced effects on VSMC calcifications and MMP upregulations involve TCF-sites, we repeated the previous experiments in the presence of a specific β-Catenin/TCF site-inhibitor. As expected, this inhibition of Wnt signaling blocked the Wnt agonist I-induced Wnt activation (Fig. 3a) and the calcification of VSMC cultures (Fig. 3b). Further, the Wnt agonist-induced elevation of gelatinolytic activities in VSMC supernatants were attenuated by specific inhibition of Wnt signaling (Fig. 3c).

Direct effects of the Wnt inhibitor on the enzymatic activities of the gelatinases MMP-2 and MMP-9 were excluded in fluorogenic MMP activity measurements (Additional file 1: Figure S2).

### Wnt antagonism reduces CM-induced transcription of MMPs and the calcification of VSMC cultures

**Fig. 4:**
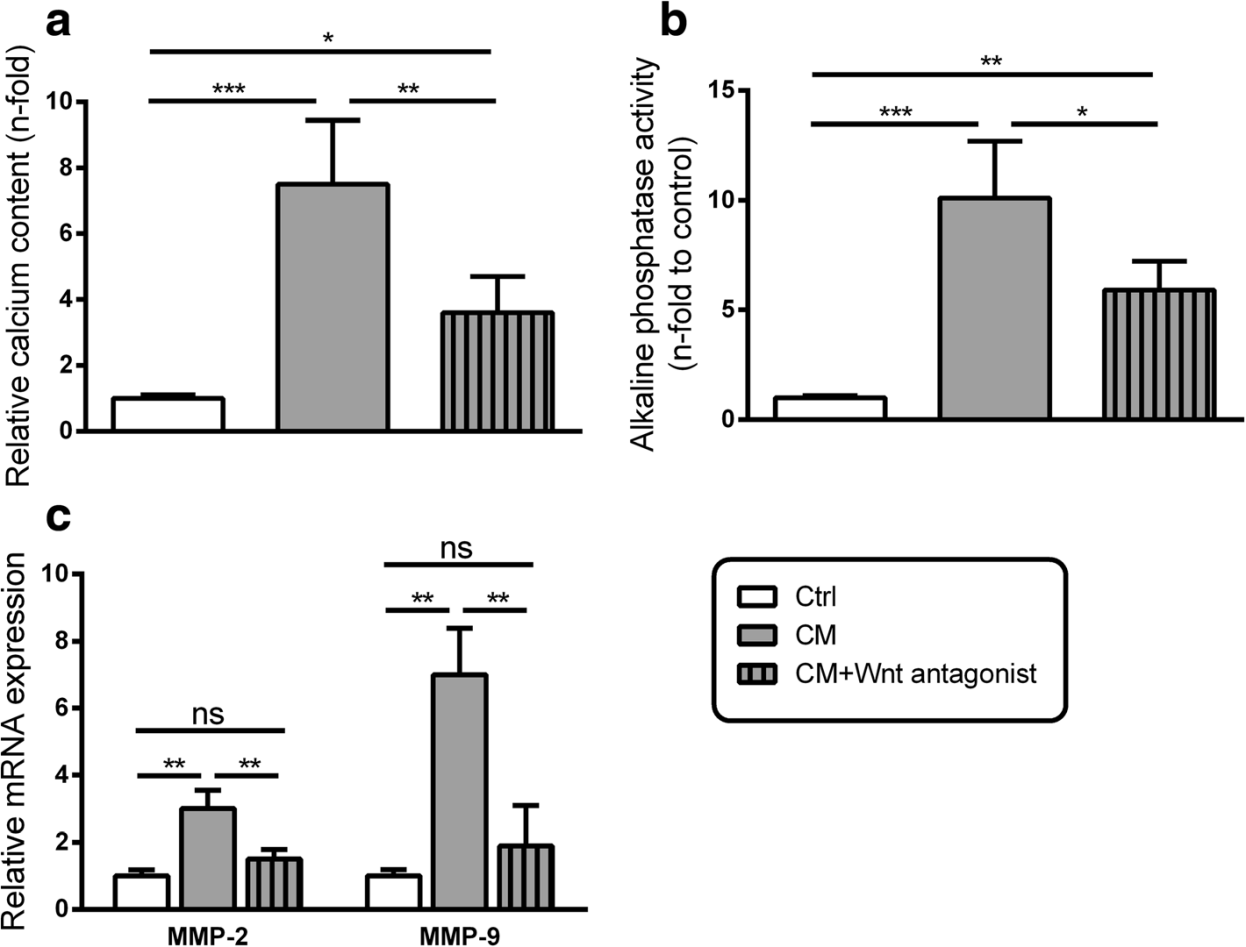
Effects of Wnt antagonism on CM-induced VSMC calcification and MMP transcription in VSMC. VSMC were treated as indicated. Measurements of calcium contents (**a**) or alkaline phosphatase (ALP) activities (**b**) in VSMC cultures served as markers for VSMC calcifications (*n* = 4). Shown are means ± SD. ^ns^not significant, *P ≤ 0.05, **P ≤ 0.01, ***P ≤ 0.001. **c** VSMC were treated for 24 h with CM with or without the presence of the Wnt antagonist FH535. The effects on mRNA expressions of MMP-2 and MMP-9 were determined by qPCR measurements (*n* = 3) Shown are means ± SD, nsnot significant, *P ≤ 0.05, **P ≤ 0.01, ***P ≤ 0.001

The preceding experiments showed that CM induced both, calcification and Wnt-signaling in VSMC. In addition, a Wnt-agonist (to a lesser extent than CM) also induced VSMC calcification. To verify these results, we therefore finally investigated whether Wnt antagonism interferes with CM-mediated effects in VSMC. Indeed, Wnt antagonism significantly reduced the CM-induced calcification of VSMC cultures. However, the measured calcium and alkaline phosphatase contents were still significantly upregulated compared to control treated cells (Fig. 4a, b). Taking into account the results from Figs. 1a and 2a, we assumed that this interference of Wnt antagonism with CM-induced calcification involves the alteration of MMP transcription in VSMC. Indeed, the presence of the Wnt-antagonist significantly reduced the CM-induced upregulation of MMP-2 and in particular of MMP-9 in VSMC (Fig. 4c).

## Discussion

Recent studies have shown that aberrant Wnt signaling is involved in vascular remodeling and calcification [17, 19]. We recently demonstrated an essential role of MMP-2 and MMP-9 in the phenotypic transition of VSMC and the development of uremic vascular calcification [15]. In the present study, we demonstrate a functional link between Wnt signaling and MMP expression in the process of calcification of VSMC.

Pro-calcifying culture conditions induced Wnt signaling in VSMC and Wnt agonist-induced Wnt signaling stimulated VSMC to calcify even under normal (non-calcifying) culture conditions. This clearly points to the fact that the Wnt pathway in VSMC contributes to calcifications.

The Wnt-mediated effects in VSMC included the induction of gene expressions of the gelatinases MMP-2 and MMP-9 which were recently shown to be strong promoters of VSMC calcifications [15]. In addition, the pro-calcifying culture conditions stimulated the transcription of Wnt5a which is a non-canonical Wnt protein known to promote osteogenesis [20]. This points to a transcriptionally regulated calcium/phosphorous-induced Wnt-activation in VSMC which was also shown by other studies where elevated phosphate concentrations promoted β-catenin signaling [21].

Also a Wnt-induced MMP expression has been described earlier, even if only in immune cells [22]. All the above mentioned effects of Wnt signaling on calcification and MMP expression were reduced by specific Wnt antagonism via the TCF-site, suggesting a functional link between Wnt signaling and MMP expression during vascular calcification.

Interestingly, simultaneous inhibition of MMP-2 and MMP-9 reduced a CM-induced Wnt activation in VSMC which suggested direct effects of MMPs on Wnt signaling in VSMC. However, this assumption was disproved by subsequent experiments showing that recombinant MMPs had no direct effects on Wnt activation in VSMC after 24–48 h.

Nevertheless, a link between enhanced proteolytic activity and enhanced Wnt signaling was brought up by the fact that recombinant MMPs induced a (time-delayed) activation of Wnt signaling in VSMC cultures after 72 h which could by blocked by MMP-inhibition. One explanation might be the generation of Wnt-stimulating matrix fragments (such as elastin fragments) in VSMC cultures due to enhanced proteolytic activity [11, 23]. This issue demands further research in follow up studies.

A final set of experiments showed inhibitory effects of Wnt antagonism on CM-induced VSMC calcification, thus, confirming the contribution of Wnt signaling to VSMC calcification. Wnt antagonism also reduced CM-induced MMP expressions in VSMC which confirmed that MMPs are regulated downstream of Wnt signaling during VSMC calcification.

## Conclusions

In summary, we here demonstrate that calcification inducing culture conditions (elevated calcium and phosphorous levels) induce Wnt activation in VSMC which then leads to enhanced expression of MMP-2 and MMP-9. Thus, the present study and our previously published data suggest the following hypothesis: in CKD, elevated serum levels of phosphorus and/or calcium (amongst others) may induce vascular calcifications by enhancing Wnt activity in VSMC which entails enhanced MMP production. The MMPs then further promote the process of calcification [15].

We conclude that suitable inhibitors of Wnt signaling and of MMPs might be of therapeutic value in the treatment of vascular calcifications. Thus, they should be applied alone or in combination to experimental models of CKD-associated vascular calcifications to verify if our in vitro data are of relevance in vivo.

## Additional file

Additional file 1: Figure S1. It shows that direct effects of the MMP inhibitors on Wnt signaling in VSMC could be excluded. **Figure S2.** It shows that direct effects of the Wnt inhibitor on the enzymatic activities of the gelatinases MMP-2 and MMP-9 in VSMC could be excluded. (TIF 186 kb)
